# A Comparative Analysis of Innate Immune Responses and the Structural Characterization of Spike from SARS-CoV-2 Gamma Variants and Subvariants

**DOI:** 10.3390/microorganisms12040720

**Published:** 2024-04-02

**Authors:** Aline Miranda Scovino, Elizabeth Chen Dahab, Israel Diniz-Lima, Etiele de Senna Silveira, Shana Priscila Coutinho Barroso, Karina Martins Cardoso, Dirlei Nico, Gustavo José Makhoul, Elias Barbosa da Silva-Junior, Celio Geraldo Freire-de-Lima, Leonardo Freire-de-Lima, Leonardo Marques da Fonseca, Natalia Valente, Valeria Nacife, Ana Machado, Mia Araújo, Gustavo Fioravanti Vieira, Alex Pauvolid-Corrêa, Marilda Siqueira, Alexandre Morrot

**Affiliations:** 1 Instituto de Microbiologia, Universidade Federal do Rio de Janeiro, Rio de Janeiro 21941-902, Brazilecdahab@gmail.com (E.C.D.); dirlei@micro.ufrj.br (D.N.); 2 Laboratório de Imunoparasitologia, Fundação Oswaldo Cruz (Fiocruz), Rio de Janeiro 21040-360, Brazil; 3 Instituto de Biofísica Carlos Chagas Filho, Universidade Federal do Rio de Janeiro, Rio de Janeiro 21941-902, Brazil; israel@biof.ufrj.br (I.D.-L.); gustavoj.makhoul@gmail.com (G.J.M.); eliasbarbosa@biof.ufrj.br (E.B.d.S.-J.); celio@biof.ufrj.br (C.G.F.-d.-L.); leolima@biof.ufrj.br (L.F.-d.-L.);; 4 Programa de Pós-Graduação em Genética e Biologia Molecular, Universidade Federal do Rio Grande do Sul (UFRGS), Porto Alegre 91501-970, Brazil; etielesenna@gmail.com (E.d.S.S.);; 5 Laboratório de Biologia Molecular, Instituto de Pesquisa Biomédica, Hospital Naval Marcílio Dias, Marinha do Brazil, Rio de Janeiro 20725-090, Brazil; shanapriscila@gmail.com (S.P.C.B.); kmcardoso@bioqmed.ufrj.br (K.M.C.); 6 Biomanguinhos, Fundação Oswaldo Cruz (Fiocruz), Rio de Janeiro 21040-900, Brazil; 7 Curso de Medicina, Universidade Castelo Branco (UCB), Rio de Janeiro 21710-255, Brazil; 8 Laboratório de Vírus Respiratórios e Sarampo, COVID-19 National Reference Laboratory of Brazil and World Health Organization COVID-19 Reference Laboratory, Instituto Oswaldo Cruz, Fundação Oswaldo Cruz (Fiocruz), Rio de Janeiro 21040-360, Brazil; natalia.valente02@gmail.com (N.V.); vnacife@hotmail.com (V.N.); bbbiaml@gmail.com (A.M.); pauvolid-correa@ufv.br (A.P.-C.);; 9 PPGSDH—Programa de Pós-Graduação em Saúde e Desenvolvimento Humano, Universidade La Salle, Canoas 92010-000, Brazil; 10 Department of Veterinary Integrative Biosciences, Texas A&M University, College Station, TX 77843, USA; 11 Laboratório de Virologia Veterinária de Viçosa, Departamento de Veterinária, Universidade Federal de Viçosa, Viçosa 36570-900, Brazil; 12 Escola de Medicina, Universidade Federal do Rio de Janeiro, Rio de Janeiro 21941-909, Brazil

**Keywords:** COVID-19, SARS-CoV-2, P.1 variant, B.1 strain, cytokines, mononuclear cells, neutrophils

## Abstract

The SARS-CoV-2 P.1 variant, responsible for an outbreak in Manaus, Brazil, is distinguished by 12 amino acid differences in the S protein, potentially increasing its ACE-2 affinity and immune evasion capability. We investigated the innate immune response of this variant compared to the original B.1 strain, particularly concerning cytokine production. Blood samples from three severe COVID-19 patients were analyzed post-infection with both strains. Results showed no significant difference in cytokine production of mononuclear cells and neutrophils for either variant. While B.1 had higher cytopathogenicity, neither showed viral replication in mononuclear cells. Structural analyses of the S protein highlighted physicochemical variations, which might be linked to the differences in infectivity between the strains. Our studies point to the increased infectivity of P.1 could stem from altered immunogenicity and receptor-binding affinity.

## 1. Introduction

The severe acute respiratory syndrome coronavirus 2 (SARS-CoV-2), a member of the *Betacoronavirus* family, is the causative agent for COVID-19 [1]. First identified in China in 2019, SARS-CoV-2 rapidly spread worldwide, leading to a pandemic. SARS-CoV-2 has given rise to several variants of concern (VOCs), including B.1.1.7 (Alpha), B.1.351 (Beta), P.1 (Gamma), B.1.617.2 (Delta) and B.1.1.529 (Omicron) [2]. The clinical manifestations of COVID-19 range from mild symptoms like cough, fever, fatigue and nausea, to severe conditions such as thrombosis, the multisystem inflammatory syndrome in children (MIS-C) and acute respiratory distress syndrome (ARDS) [3,4,5,6]. The infection triggers a cytokine storm orchestrated by lung-recruited macrophages and neutrophils, and is characterized by a marked release of cytokines like IL-6, IL-5, IL-10, IL-12p70, IL-17 and IFN-y [4,5].

SARS-CoV-2 has positive-sense single-stranded RNA genomes spanning around 29 kb [7]. It carries distinct structural proteins, including spike (S) protein, envelope (E), membrane (M) and nucleocapsid (N). Binding of the S protein to the ACE2 receptor, present in various mammalian tissues, is a crucial initial step in coronaviruses’ entry process, including SARS-CoV-2 [8]. This entry mechanism involves a two-step process: firstly, the viral spike (S) protein must attach to a cell surface receptor, and subsequently, it requires cleavage by a cellular protease to enable membrane fusion. This process depends on both an appropriate receptor and a host-encoded protease capable of cleaving the S protein near the receptor-binding site.

The S protein comprises two functional domains: S1, housing the receptor-binding domain (RBD) responsible for engaging ACE2, and S2, facilitating viral particle fusion with the cell membrane. TMPRSS2, a host cell surface serine protease, cleaves the S protein, enabling viral entry [9]. Notably, three proteins (ACE, THOP1 and NLN) share structural similarities with ACE2, despite a low percentage of identical amino acid residues. These proteins possess crucial local secondary structures for interacting with the RBD of the SARS-CoV-2 spike protein, including specific helices and beta-sheets [10].

Moreover, an analysis of protein expression across various tissues reveals that THOP1 exhibits a higher expression than ACE2, particularly in the lung, colon, esophagus mucosa and various brain compartments, where ACE2 expression is comparatively lower. This highlights THOP1’s potential as an alternative SARS-CoV-2 receptor, with significant implications for understanding viral entry mechanisms, tissue-specific effects and potential associations with the immune response and neurological manifestations in COVID-19 [10].

In summary, the primary determinants of SARS-CoV-2’s entry into human cells involve the ACE2 receptor and TMPRSS2 protease, with the potential alternate receptor, CD147 (BSG), also known as Basignin, under consideration. Furthermore, other factors, including ANPEP, CD209, CLEC4G/M and DPP4, previously shown to facilitate entry for other coronaviruses, are emerging as potential facilitators for SARS-CoV-2’s entry. CD26 (DPP4), expressed across various cell types except B cells, may contribute to systemic virus dissemination, particularly within the lungs. Highly expressed in both epithelial tissues and innate and adaptive immune cells, CD147 (BSG) raises the possibility of immune cell infection or viral carriage, potentially contributing to local and systemic viral spread and immune responses [11].

Furthermore, various cellular proteases, including TMPRSS4, TMPRSS11A/B, furin and cathepsins (CTSL/B), can serve as alternative priming factors for viral entry. Additionally, recognized restriction factors (RFs), such as LY6E and IFITM1-3, provide defense against SARS-CoV-2’s entry. Post-entry processes, such as viral genome replication, depend on factors such as TOP3B and MADP1. Additionally, proteins involved in viral assembly and intracellular trafficking, including RHOA, RAB10, RAB14, RAB1A, AP2A2, AP2M1 and CHMP2A, are being considered as potential modulators of SARS-CoV-2’s entry and replication within human cells. Together, these factors play pivotal roles in determining the virus’s cellular tropism and infection mechanisms [11].

Subsequently, Open Reading Frames (ORFs) in the genome are translated. ORF1a and ORF1b encode polyproteins pp1a and pp1ab, which are cleaved into 16 nonstructural proteins (NSPs) involved in virus transcription and replication [12]. 

While Type I and III Interferons (IFNs) normally respond rapidly to viral infections, SARS-CoV-2 infection delays the release and production of IFNs [13]. This imbalance in the IFN response could disrupt viral suppression regulation, resulting in an increased production of inflammatory cytokines and triggering a hyperinflammation [13,14] reaction which characterizes the cytokine storm [15]. In individuals progressing to severe COVID-19, a notable rise in the neutrophil/lymphocyte ratio is often observed [16]. In critically ill patients, inflammatory monocytes have been implicated in the production of greater amounts of proinflammatory cytokines, which is a hallmark of the cytokine storm responsible for ARDS [3]. IL-17, specifically in the context of ARDS, promotes lung tissue degradation by recruiting neutrophils, generating pro-inflammatory mediators and inhibiting apoptosis through granulocyte colony-stimulating factor (G-CSF) expression [17]. The P.1 variant carries significant mutations—K417T, E484K and N501Y—within its RBD, leading to an enhanced binding affinity of ACE2. This characteristic facilitates the viral entry into host cells, as well as replication [18,19,20]. Specifically, the K417T and E484K mutations interact with ACE2 [21].

In a study conducted by Turoňová et al., they revealed that structural changes in the spike receptor-binding domain (RBD) are crucial for understanding how new virus variants can evade antibodies. In its prefusion state, the spike protein is heavily glycosylated, forming a protective glycan shield. The RBDs can adopt either an upward or downward orientation, with the upward position favoring interactions with host cells. The stalk region connecting the spike head to the viral membrane is highly dynamic, featuring flexible hinge regions that allow significant movement. These structural characteristics collectively contribute to the virus’s ability to evade antibody recognition, emphasizing the importance of understanding these conformational changes in the context of evolving SARS-CoV-2 variants. These findings suggest that the spike protein’s flexibility, orientation and glycan shield are key factors in immune evasion. This knowledge is valuable for developing neutralizing antibodies and vaccines. Additionally, the authors propose exploring pharmaceutical agents targeting the spike protein’s hinge regions to disrupt conformational changes and ACE2 receptor interactions, potentially offering antiviral therapies against SARS-CoV-2 [22,23]. 

These RBD mutations can significantly influence viral binding and neutralization, potentially affecting vaccine efficacy [24]. In particular, individuals vaccinated with BNT162b2 (Pfizer/BioNTech) show reduced effectiveness against the P.1 variant compared to those who have previously recovered from COVID-19 [25]. By the end of 2020, the P.1 variant was first identified in a traveler from the Brazilian state of Amazonas to Japan [26,27,28]. A drastic increase in the number of P.1 variant cases, and consequently in the number of deaths, this persistent transmission of SARS-CoV-2 is closely linked to the prevalence of the P.1 variant and the emergence of subvariants, some of which exhibit increased transmissibility due to NTD deletions or mutations in the S protein, particularly within the furin cleavage region [29]. Subvariants within these mutations, named P.1.6 and P.1.7 (P681H) as well as P.1.8 (P681R), have been identified [29]. Additionally, the P.1.3 variant is characterized by a deletion in the NTD region, and the P.1.4 and P.1.5 variants are marked by the N679K mutation [29]. Most of the genetic alterations identified in the P.1 subvariants also appear in other VOCs (B.1.1.7, B.1.617.2 and B.1.1.529), and are associated with increased viral infectivity, immune escape or both [29,30,31]. Notably, the P681R mutation enhances viral replication, fusion and cell-to-cell spread in vitro [32,33,34]. 

Emerging SARS-CoV-2 variants, including Omicron and B.1.351, have heightened concerns over potential evasion of vaccine-induced antibodies. The Omicron variant, likened to a ship’s stern, hull, bow and rudder for its nuanced RBD regions, suggests altered interactions with antibodies, prompting questions about the current vaccines’ effectiveness. The B.1.351 variant, first identified in South Africa, harbors mutations such as K417N, E484R and N501Y on its spike protein. These changes raise alarms about the efficacy of prominent vaccines, including Pfizer-BioNTech and Oxford–AstraZeneca. Notably, while Janssen and Oxford–AstraZeneca vaccines have indicated reduced efficacy against B.1.351, the full ramifications for the Pfizer-BioNTech vaccine remain under investigation. Meanwhile, other variants (such as B.1.1.7) also present challenges, with certain studies suggesting that specific monoclonal antibodies might be less effective against them. The presence of the D614G mutation in many strains has been associated with increased infectivity. Vaccines, although still protective, may see varied efficacies against these strains. Testing using pseudo-viruses or SARS-CoV-2 with mutations in the spike protein’s RBD revealed that the antibody response in vaccinated individuals might be weaker in preventing initial infections in areas such as the nasal and oral cavities [35,36,37].

This scenario prompted the inquiry of whether the newly prevalent variant in Manaus could exhibit increased pathogenicity compared to the original Wuhan variant, manifesting in increased mortality and transmission. Several studies have unveiled distinct biological and clinical attributes among the emergent variants, setting them apart from both each other and the original Wuhan strain (B.1). Enhanced pathogenicity has been associated with variants such as B.1.1.7, B.1.351, B.1.617.2 and P.1, leading to an escalation of severe cases and fatalities [38,39,40,41,42,43,44,45,46,47]. Conversely, the B.1.1.529 variant exhibits an opposing trend [48,49,50,51,52,53,54].

While the data regarding the pathogenicity of the P.1 variant remain enigmatic, certain studies have hinted at unique epidemiological outcomes. Elevated mortality and transmissibility have been suggested, underscoring potential biological distinctions in comparison to the B.1 strain [27,47,55]. Given the critical roles played by neutrophils [56]. and mononuclear cells [57] in the pathogenesis of severe COVID-19, the main goal of our study was to evaluate whether the P.1 variant exhibits a higher pathogenic biological potential when compared to the B.1 strain in terms of their interactions with mononuclear cells, neutrophils and cytokine stimulation. Neutrophils and mononuclear cells obtained from severe COVID-19 patients were experimentally infected by both the B.1 strain and the P.1 variant. We devised a model in which we could evaluate cytokine production after both cells are equally stimulated with both viruses, mimicking a situation where infection routes are bypassed, and the virus-to-cell ratio is the same. Additionally, we assessed the physicochemical characteristics of the S protein of the P.1 variant, its subvariants and the B.1 strain through bioinformatics analysis. These assessments may help corroborate possible biological differences among them, as observed in the P.1 epidemic, such as greater infectivity and transmissibility.

## 2. Materials and Methods

### 2.1. Human Samples

Blood samples were obtained from three patients with severe COVID-19 hospitalized during the acute phase of infection. Cases of acute SARS-CoV-2 infection were confirmed upon positive results of nucleic acid sequencing with real-time RT-qPCR from nasopharyngeal swab samples, utilizing FDA-approved RNA testing. The severity of patients with COVID-19 was clinically classified by fever, respiratory infection, a respiratory rate of 23 breaths per minute, dyspnea and oxygen saturation < 93% at room air. This study was approved by the Research Ethics Committee (CEP) of the Brazilian National Health Council, and all patients provided informed consent in accordance with relevant ethical regulations and current legislation. This study was conducted at the Hospital Naval Marcílio Dias (CAAE # 31642720.5.0000.5256). 

### 2.2. Virus Strains

Mononuclear cells and neutrophils were infected with two severe acute respiratory syndrome coronavirus 2 (SARS-CoV-2) lineages, B.1 (hCoV-19/Brazil/RJ-FIOCRUZ-314/2020, GISAID accession number EPI_ISL_414045) and P.1 (hCoV-19/Brazil/AM-FIOCRUZ-3521-1P/2021, EPI_ISL_1402431), within a Biosafety Level 3 (BSL-3) facility for all experiments.

### 2.3. Cell Cultures

Mononuclear cells and neutrophils were isolated from a 20 mL collection of heparinized peripheral blood obtained from both patients with severe COVID-19 and healthy donors. The collected blood was gently layered into a 50 mL tube containing a 1:2 ratio of Ficoll, and the gradient was centrifuged at 400× *g* for 30 min at room temperature, without braking or acceleration. After centrifugation, the upper portion containing mononuclear cells was carefully retrieved for subsequent cytokine, microscopy, and viral replication assays. The lower portion containing neutrophils was collected using a Pasteur pipette. Following lysis of red blood cells, the granulocytes were resuspended in RPMI medium, counted and adjusted for each experimental condition involving microscopy and cytokine assays. Similarly, mononuclear cells were counted and adjusted for each experimental condition, followed by their placement in a 24-well plate containing circular coverslips at the bottom of each well. After a 2 h incubation period in RPMI medium supplemented with 1% nutridoma, non-adherent cells were removed, and the medium was replaced to proceed with the subsequent assays.

### 2.4. Microscopy Assay

Mononuclear cells that adhered to coverslips were suspended in RPMI medium containing 1% nutridoma and adjusted to a concentration of 1 × 10^5^ mononuclear cells/mL. These cells were then treated with SARS-CoV-2 B.1 virus at a multiplicity of infection (MOI) of 9.0, as well as SARS-CoV-2 P.1 virus at MOI 9.0. Mock controls were established by culturing with RPMI medium. Subsequently, the samples were incubated at 37 °C/5% CO_2_ for 24 h. Following the incubation period, the coverslips were carefully washed with PBS and then fixed in 4% paraformaldehyde for 20 min. The coverslips were then delicately removed from the plates and subjected to staining using the panoptic method (Laborclin), which is based on the May–Grünwald–Giemsa technique. These stained coverslips were then mounted on slides using Entellan.

Mononuclear cells and neutrophils were enumerated under an optical microscope at a magnification of 1000×. For each patient, we utilized two coverslips for each treatment, with three patients in total. This resulted in 6 coverslips for the mock group, 6 for infection with P.1 and 6 for infection with B.1. For each coverslip, 20 photographs of unique visual fields were captured. In other words, for each treatment, a total of 120 fields were analyzed. In the case of the mock group, we quantified the number of cells per photographed field and computed the average across the three patients. This average was used to assess the percentage of cells relative to the mock group, which we considered as 100%. Subsequently, we calculated the percentage reduction or increase of cells (using the rule of three) for each of the 20 fields from the three patients infected with P.1 or B.1. 

For the calculation of absolute cell numbers, we determined the average percentage from the 20 visual fields of each coverslip. The cell count was then derived by accounting for the percentage of the total number of cells initially placed on each slide for the tests (1 × 10^5^). Therefore, each point on the graph corresponds to a coverslip.

### 2.5. Detection and Quantification of Cytokines

The supernatants obtained from the cultures of mononuclear cells and neutrophils under the same experimental conditions as the microscopy assay were carefully collected and subsequently stored at −80 °C in a freezer for subsequent analysis. Following the manufacturer’s guidelines (Quantikine Elisa/R&D System, Minneapolis, MN, USA), each sample was plated in 96-well plates for the quantification of the following cytokines: MIP-1β, IL-6, IFN-γ, IL-1ra, IL-5, RANTES, IL-1β, bFGF, PDGF-BB, IL-4, MCP-1, MIP-1α, IL-10, G-CSF, IL-12p70 and IL-17A. 

The concentrations of the supernatants were then plotted for each experimental group.

### 2.6. Detection and Quantification of SARS-CoV-2 with RT-PCR

In this study, mononuclear cells (3 × 10^5^ cells/well) from healthy donors were cultivated in a 24-well plate. Subsequently, these cells were exposed to infections caused by both the P.1 and B.1 lineages of the SARS-CoV-2 virus. A Multiplicity of Infection (MOI) of 0.01 was employed for the viral infection in both lineages. As a control, another set of cells was inoculated with PBS 1X. 

The mononuclear cells were then incubated for 24, 48 or 72 h. After that, the supernatants were harvested for reverse transcription polymerase chain reaction (RT-PCR) analysis. RNA extractions were carried out using the QIAamp Viral RNA Mini Kit (QIAGEN, Hilden, Germany), and subsequently, RT-PCR was performed utilizing the Molecular SARS-CoV [58].

### 2.7. Modeling

The reference sequence for WT-Wuhan was derived from the P0DCT2 sequence (Uniprot). Information pertaining to the specific positions and mutations present in the variants was sourced from the Global Initiative on Sharing All Influenza Data (GISAID) database [52]. To construct the variant sequences, mutations observed in at least 90% of the sequences available in the GISAID dataset were incorporated.

The search within the GISAID database was executed using the Lineage Comparison tool, employing the subsequent parameters: Selected lineages = Gamma (including sublineages), and an additional specific lineage was added, namely B.1.1.28. This search strategy facilitated the identification of the mutations occurring in the strains, thereby enabling the necessary modifications to be made to the S protein sequence.

It is noteworthy that the P.1.5 variants share the same mutations as P.1.4, while the P.1.6 variant exhibits identical mutations to those of P.1.7. Consequently, images representing these structures were omitted from the figures. To accurately position the mutations, we employed Jalview. The resulting mutated FASTA sequences were modeled using the structure 7KRQ from the RCSB Protein Data Bank as a template in the Swissmodel platform. Subsequently, the quality of the generated models was assessed through the PROCHECK analysis.

### 2.8. Calculation of Electrostatic Potential and Solvent-Accessible Surface

The models produced for each variant were employed to conduct analyses encompassing electrostatic surface potential, hydrophobicity and solvent-accessible area calculations. To generate the surface electrostatic potential map, the Adaptive Poisson–Boltzmann Solver (APBS) was utilized, accessible at https://server.poissonboltzmann.org (accessed on 2 November 2020). Visual representations containing information about mutation positions within the crystals, as well as details concerning surface electrostatic potential and hydrophobicity, were crafted employing ChimeraX version 1.4.

For the determination of solvent-accessible surface area, the GetArea tool (Fraczkiewicz et al., 1998 [59]) was employed. The gathered information for each model was subsequently saved, and cluster analysis was performed using the pvclust package within RStudio version 4.2. A comparative assessment of interaction properties among the modeled proteins was conducted using the webPIPSA tool. 

Utilizing the data from the electrostatic map, surface images were generated with the aid of ChimeraX. Additionally, the generated models underwent scrutiny through GetArea to identify solvent-accessible areas and assess the hydrophobicity of the residues.

### 2.9. Figures with the Epitope Location Frequencies (Immunogenicity Scale)

For further insight, the models were subjected to clustering based on residue data for solvent-accessible surface area (ASA) and hydrophobicity, achieved through the GetArea tool. This clustering was performed using the pvclust package in the R programming environment.

For the graphical representation of the figures, images of the models generated in ChimeraX were utilized. These models were superimposed, and the calculation of the electrostatic surface was conducted using the map generated with APBS. This approach allowed for the visualization of distinct characteristics of each model concerning both the RBD region and the lateral orientation. In addition, images illustrating the hydrophobic surface features were generated at corresponding positions.

In the context of the variants, mutations were denoted by a pink color, while the cleavage site region was highlighted in orange. The correlation between the variants and their electrostatic surfaces was evaluated using the webPIPSA program. It is important to note that webPIPSA assesses and compares the electrostatic potential of proteins, generating a clustering of the analyzed models and an epogram accompanied by a color-coded distance matrix.

The Immunome Browser tool, accessible through the Immune Epitope Database and Analysis Resource (IEDB) at www.iedb.org, was employed to retrieve the figures illustrating the experimentally described immunogenicity of the SARS-CoV-2 S protein. The search parameters utilized for this purpose were as follows: assay type—B cell, epitope source—organism = SARS-CoV-2 (ID2697049), antigen—spike Glycoprotein P0DTC2, MHC restriction—no MHC assays, host—human.

### 2.10. Statistical Analysis

Results are presented as the mean ± standard error of the mean (SEM), and statistical significance was determined at a threshold of *p* ≤ 0.05. Statistical comparisons were carried out with respect to the control group using Student’s *t*-test. Multiple comparisons among the experimental groups were conducted using a two-way analysis of variance (ANOVA) followed by Dunnett’s test for post hoc analysis. All statistical analyses were performed using GraphPad Prism 6.0 software.

## 3. Results

### 3.1. Both Variants Do Not Change the Cytokine Profile of Mononuclear Cells and Neutrophils

The cytokine storm, driven by macrophages and neutrophils, constitutes a critical factor that contributes to the severity of COVID-19 [1,3,4,5]. Thus, the potential of variants to elicit cytokine production holds implications for their pathogenicity. Notably, the cytokine storm is primarily orchestrated by lung tissue-resident cells, such as epithelial and macrophage populations, alongside stromal cells, being further exacerbated by the recruitment of mononuclear cells and neutrophils into the tissue [60]. In this context, our investigation focused on deciphering the cytokine spectrum released by mononuclear cells and neutrophils upon an interaction with SARS-CoV-2.

**Figure 1:**
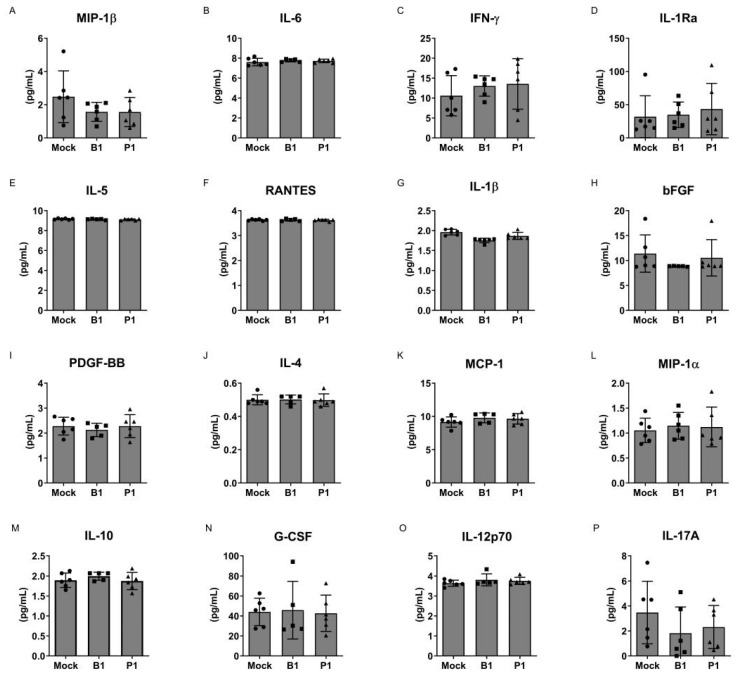
Cytokine profile of SARS-CoV-2-treated mononuclear cells. Cytokine analysis of the supernatant from non-treated mononuclear cells (mock group), mononuclear cells treated with the B.1 strain (B1) and mononuclear cells treated with the P.1 variant (P1). (**A**) MIP-1β, (**B**) IL-6, (**C**) IFN-γ, (**D**) IL-1Ra, (**E**) IL-5, (**F**) RANTES, (**G**) IL-1β, (**H**) bFGF, (**I**) PDGF-BB, (**J**) IL-4, (**K**) MCP-1, (**L**) MIP-1α, (**M**) IL-10, (**N**) G-CSF, (**O**) IL-12p70 and (**P**) IL-17A. No significant differences were observed between the groups. Statistical comparisons were performed against the control group using the Student’s *t*-test. Multiple comparisons among the experimental groups were conducted using two-way analysis of variance (ANOVA) followed by Dunnett’s test.

**Figure 2:**
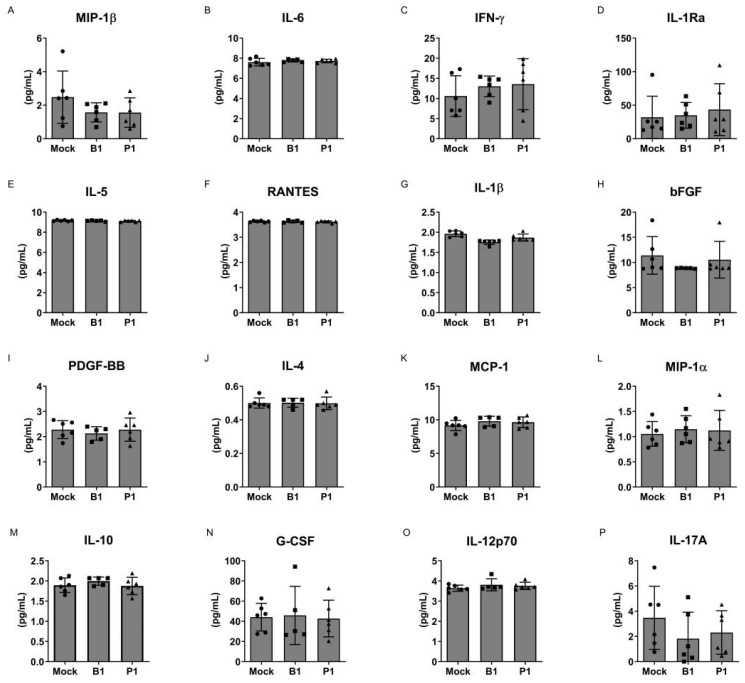
Cytokine profile of SARS-CoV-2-treated neutrophils. Cytokine analysis of the supernatant from non-treated neutrophils (mock group), neutrophils treated with the B.1 strain (B1) and neutrophils treated with the P.1 variant (P1). (**A**) MIP-1β, (**B**) IL-6, (**C**) IFN-γ, (**D**) IL-1Ra, (**E**) IL-5, (**F**) RANTES, (**G**) IL-1β, (**H**) bFGF, (**I**) PDGF-BB, (**J**) IL-4, (**K**) MCP-1, (**L**) MIP-1α, (**M**) IL-10, (**N**) G-CSF, (**O**) IL-12p70 and (**P**) IL-17A. No significant differences were observed between the groups. Statistical comparisons were performed against the control group using the Student’s *t*-test. Multiple comparisons among the experimental groups were conducted using two-way analysis of variance (ANOVA) followed by Dunnett’s test.

To address this inquiry, we utilized cells obtained from individuals severely afflicted by an acute COVID-19 infection. These cells were subjected to treatment with either the B.1 or P.1 variants of SARS-CoV-2 or the mock variants. After a 24 h incubation period, supernatants were collected, and cytokine production was quantified through an enzyme-linked immunosorbent assay (ELISA). Our findings consistently reveal that, across all evaluated cytokines, no significant differences emerged between mononuclear cells originating from patients treated with either the P.1 or B.1 variants, in comparison to the mock group (see Figure 1). Similarly, this uniform pattern was also reflected in the production of cytokines by neutrophils, with no discernible disparities observed when comparing the P.1 and B.1 variants (refer to Figure 2).

### 3.2. B.1 Variant Shows More Cytopathogenic than the P.1 Variant

**Figure 3:**
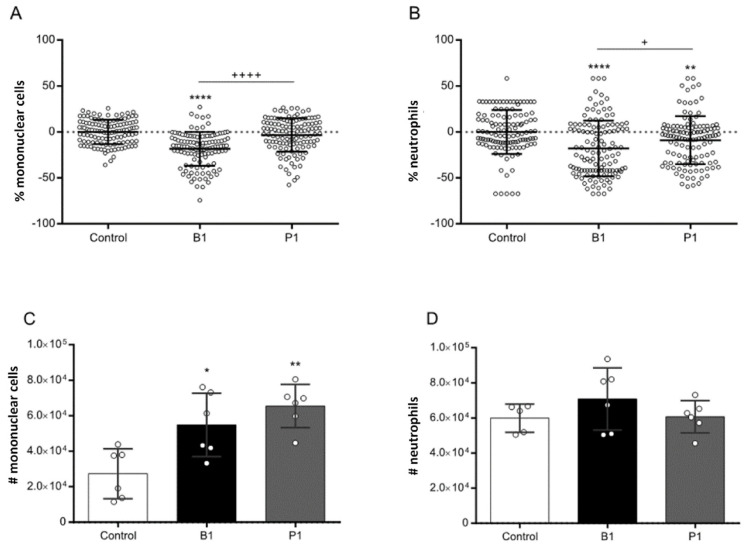
Microscopy analysis of mononuclear cell and neutrophil percentage following SARS-CoV-2 treatment. Microscopy-based analysis of (**A**,**C**) mononuclear cells and (**B**,**D**) neutrophils obtained from patients with severe COVID-19. The analysis encompasses (**A**,**C**) mononuclear cells: mock mononuclear cells, mononuclear cells infected with the b.1 variant (b1), mononuclear cells infected with the P.1 variant (P1); (**B**,**D**) neutrophils: mock neutrophils, neutrophils infected with the B.1 variant (B1), neutrophils infected with the P.1 variant (P1). (* *p* < 0.05; ** *p* < 0.01; **** *p* < 0.0001) for comparison with the mock group. (+ *p* < 0.05; ++++ *p* < 0.0001) Comparison between indicated groups. Statistical comparisons were executed against the control group using Student’s *t*-test. Multiple comparisons among the experimental groups were conducted using two-way analysis of variance (ANOVA) followed by Dunnett’s test.

Subsequently, we investigated the relative cytopathogenicity of mononuclear cells and neutrophils following the treatment with the P.1 and B.1 variants. This analysis aimed to discern whether the P.1 variant might exhibit increased cytopathogenicity, potentially correlating with escalated pulmonary inflammation and enhanced overall pathogenicity. Our experimental approach involved obtaining peripheral blood mononuclear cells and neutrophils from individuals with severe acute infections. Following a 24 h treatment period with the P.1 or B.1 variants, as well as the mock controls, we subjected the cells to panoptic staining and quantified them using light microscopy. Enumeration was performed for each visual field, enabling the derivation of both cell population percentages and absolute cell numbers (Figure 3).

Following this, we evaluated the cell death index by comparing the percentage of mononuclear cells and neutrophils treated with the B.1 and P.1 variants against the mock controls. Our results revealed a decrease in the percentage of mononuclear cells (Figure 3A) and neutrophils (Figure 3B) treated with the B.1 variant, indicating a higher level of induced cell death compared to the P.1 variant. This observation suggests that the B.1 variant may possess a greater cytopathogenic potential than the P.1 variant.

Further investigation involved the calculating the absolute counts of mononuclear cells and neutrophils after the treatment with the B.1 and P.1 variants. Armed with the cell percentages per coverslip and the overall cell count at the time of plating, we accurately estimated the absolute cell count post-infection. Strikingly, no statistically significant distinctions emerged between the absolute numbers of mononuclear cells (Figure 3C) or neutrophils (Figure 3D) following infection with either the B.1 or P.1 variant. When compared to the cell death index (Figure 3A,B), these cumulative findings suggest that the cytopathogenicity of the P.1 variant’s does not surpass that of the original B.1 variant.

### 3.3. There Is No Viral Replication within Macrophage Cells for Both SARS-CoV-2 Variants

**Figure 4:**
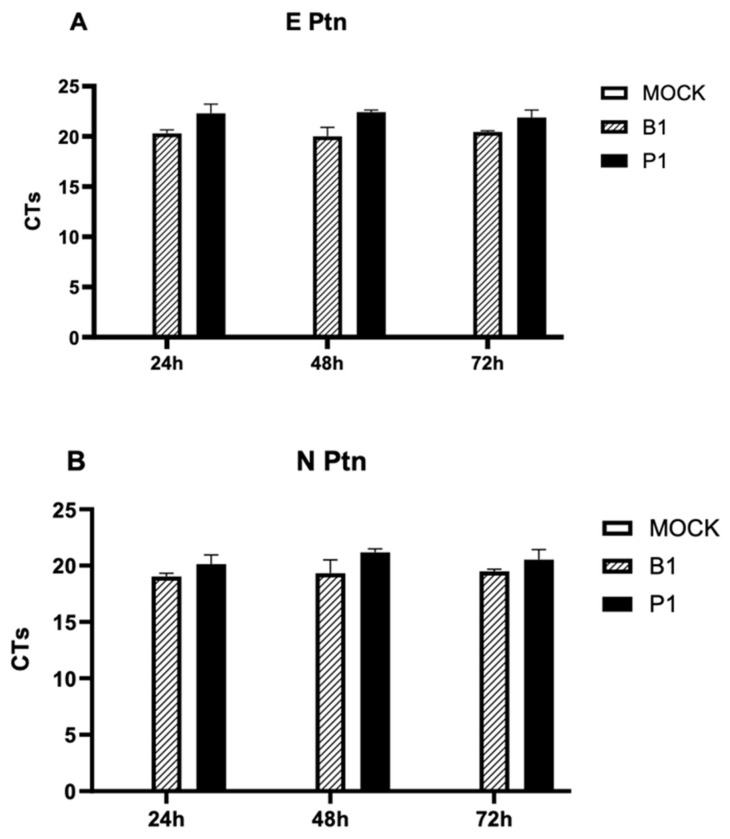
SARS-CoV-2 infection of mononuclear cells. Analysis of SARS-CoV-2 B.1 and P.1 replication cycles (cycle threshold, CT) based on (**A**) envelope genes and (**B**) nucleocapsid genes, following 24 h, 48 h and 72 h incubation periods. No statistically significant differences were observed between the groups. Statistical comparisons were executed against the control group using Student’s *t*-test. Multiple comparisons among the experimental groups were performed using two-way analysis of variance (ANOVA) followed by Dunnett’s test.

Previous research has indicated that SARS-CoV-2 is capable of infecting mononuclear cells in vitro; however, this infection does not lead to productive viral replication. In other words, these cells do not support sustained viral multiplication [61,62,63]. Considering this understanding, our focus shifted to evaluating potential disparities in the infective and replicative capabilities of the P.1 and B.1 variants (depicted in Figure 4).

Our investigation involved the isolation of mononuclear cells from healthy donors, followed by a treatment with the variants for durations of 24, 48 or 72 h. We deliberately used a low MOI of 0.01 with the intention of specifically assessing the replicative potential of each strain. Subsequently, supernatants were collected, and an RT-qPCR assay targeting the envelope (E) and nucleocapsid (N) genes was performed. Our findings indicate an absence of significant viral replication, as no discernible reduction in cycle threshold (CT) levels was observed over time, regardless of the gene region employed for detection. This collective evidence strongly suggests there is no viral replication within mononuclear cells for each of the P.1 and B.1 variants.

### 3.4. Spike Protein Structure

**Figure 5:**
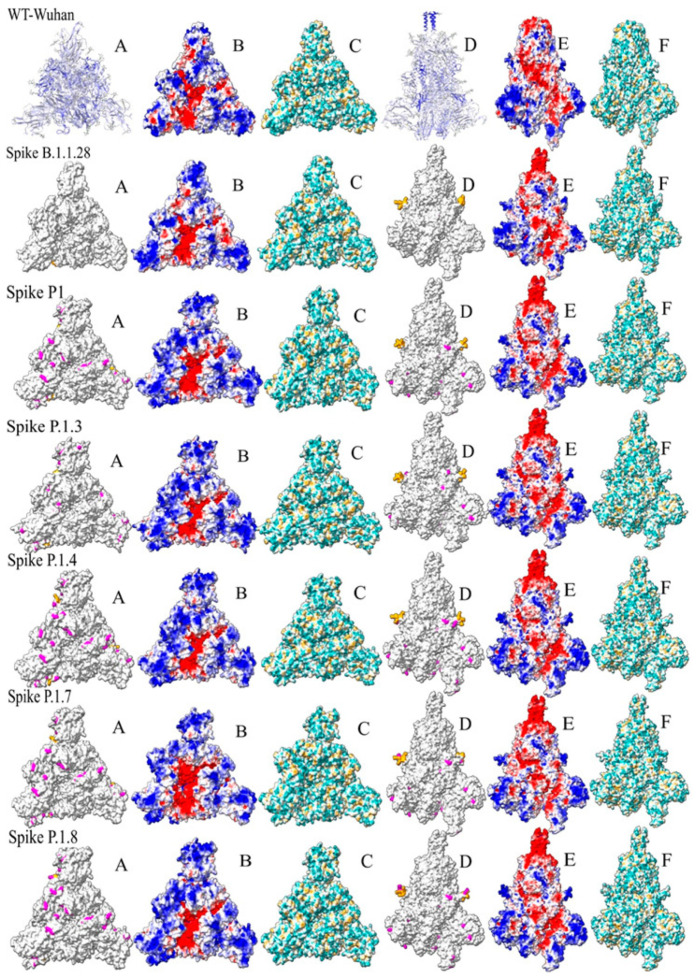
Comparative analysis of spike protein variants. This figure provides a comprehensive comparative analysis of different spike protein variants, focusing on the S Glycoprotein of SARS-CoV-2. The layout of the figure is as follows: (**A**) epitope heat map and RBD mutation map (top view): in the first line, a heat map displays normalized values of epitopes sourced from the IEDB database for the S Glycoprotein’s receptor binding domain (RBD); subsequent lines showcase RBD views of mutation positions for each variant, with cleavage sites highlighted in orange and mutations in pink. (**B**) Electrostatic potential surface (RBD view). (**C**) Hydrophobicity surface (RBD view). (**D**) Epitope heat map and mutation map (side view): In the first line, a heat map presents epitopes sourced from the IEDB database, and side view representations of the S Glycoprotein highlight mutation positions for each variant. (**E**) Electrostatic potential surface (side view). (**F**) Hydrophobic surface (side view). Each row within the figure corresponds to a specific variant. The top portion displays images associated with the Wuhan sequence. The figure allows for comparisons between variants, including P.1.3, P.1.4, P.1.7, Wuhan.1.8 and B.1.1.28. Each represented in a side-by-side position with the original spike protein information. This comparison aids in discerning differences in structure and characteristics between these variants.

The selection of spike mutations for detailed analysis in our study, as represented in Figure 5, is underpinned by the pivotal role that the spike (S) protein plays in the life cycle and infectivity of the SARS-CoV-2 virus. The S protein, specifically its receptor binding domain (RBD), facilitates the virus’s binding to the ACE-2 receptor on human cells, marking the initiation of infection. Consequently, any mutations in this region potentially alter the virus’s binding efficiency [10,11,12].

We meticulously examined the structural characteristics of the S protein, both in its wild-type form (Spike-WT) and in six distinct variants (including B.1.1.28, P.1, and its subvariants). Our focus encompassed exploring physicochemical and structural variances within immunogenic regions that prominently feature the domain responsible for interacting with the ACE-2 receptor. Notably, we evaluated parameters such as hydrophobicity, solvent-accessible surface (SAS) and electrostatic potential (EP) as key determinants of potential alterations (depicted in Figure 5).

In Figure 5, a comprehensive visual comparison of the trimeric structure of the spike protein across all analyzed variants is presented. The figure provides both top and side views of the protein structure, offering valuable insights into potential variations. Columns A, B and C: top view of the spike protein structure. Columns D, E and F: side view of the spike protein structure. Each row in the figure corresponds to a specific evaluated variant. In Column A, significant mutations for each variant are highlighted in pink, while orange marks indicate furin cleavage points. Columns B and E provide insights into the electrostatic (PE) surface of the spike proteins, while Columns C and F illustrate the hydrophobicity surface.

### 3.5. Clustering of the Analysis of ASA and Hydrophobicity of Spike Proteins

**Figure 6:**
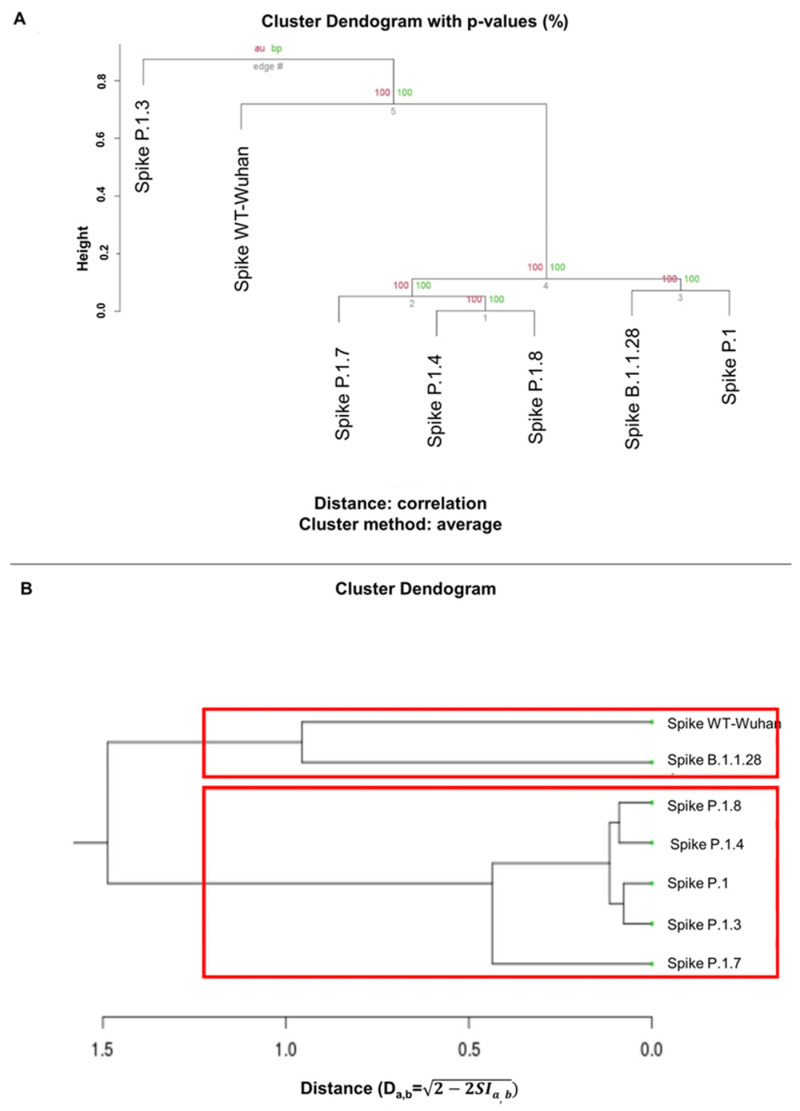
Comparative analysis of structural characteristics and clustering. (**A**) Clustering analysis of ASA and hydrophobicity: The pvclust package in RStudio was employed for clustering the analysis of residue models based on solvent-accessible surface (ASA) and hydrophobicity. Notably, the models of P.1.4, P.1.8, P.1 and B.1.1.28 exhibit smaller distances on their surfaces, and the model of P.1.7 displays a smaller distance relative to these clusters. In contrast, the P.1.3 and WT-Wuhan models clustered together, distant from the remaining analyzed models. (**B**) Cluster dendrogram of webPIPSA analysis: the webPIPSA analysis of electrostatic surfaces resulted in a cluster dendogram. This analysis reveals the clustering of the S protein from WT-Wuhan with the ancestral B.1.1.28 variant of the Gamma lineage. In contrast, the other variants are grouped together, indicating smaller distances among them. The red boxes indicates the number of clusters we determined for the analysis.

To enhance the accuracy of our analysis, we adopted a quantitative approach utilizing two distinct methodologies. We employed a comparative evaluation of the hydrophobicity and solvent-accessible surface (SAS) characteristics of the spike proteins (depicted in Figure 6A). Additionally, we examined the similarities in the electrostatic interaction properties of the proteins (illustrated in Figure 6B).

In the context of hydrophobic cluster analysis (Figure 6A), we generated clusters based on shared SAS and hydrophobicity values of the spike proteins. Notably, the wild-type virus served as an outgroup, with the P.1.3 subvariant exhibiting similarities, while B.1.1.28 clustered alongside P.1. This analysis revealed patterns suggesting that ancestral S proteins, such as WT, B.1.1.28 and P.1, share certain physicochemical characteristics, while more evolved variants occupy distinct clusters.

In the electrostatic potential (EP) cluster analysis (Figure 6B, Supplementary Figure S1), the distance matrix program yielded clusters distinguishing Spike-WT and B.1.1.28, which formed a distinct group from other variants. Notably, both P.1.7 and P.1.8 were situated in close-proximity clusters. This approach further reinforces the distinction between ancestral S proteins and their more evolved counterparts.

In both analytical approaches, our observations consistently point to shared physicochemical attributes among ancestral spike proteins (WT/B.1.1.28/P.1), while more evolved variants tend to cluster separately. These clustering insights offer valuable perspectives on potential structural and functional disparities between different spike protein variants.

## 4. Discussion

Here, our study offers an expansive examination of the impact of the SARS-CoV-2 variants, specifically B.1 and P.1, on the cytokine profiles of mononuclear cells and neutrophils (Figure 1 and Figure 2). We also explored the cytopathogenicity (Figure 3), replicative potential (Figure 4) and structural intricacies of the spike proteins of these variants.

In the context of the ongoing pandemic, understanding the implications of the novel variants is paramount. The severity of the COVID-19 disease has been directly correlated with the cytokine storm, a hyper-reactive immune response characterized by elevated levels of circulating cytokines [1,3,4,5]. It was thus crucial to ascertain if the emergent variants could influence this profile, potentially leading to augmented disease severity. Our findings, however, indicate that neither the B.1 nor the P.1 variants significantly alter the cytokine profile of mononuclear cells and neutrophils. This observation suggests that while other factors may contribute to the potential increased transmissibility or evasion from neutralization by antibodies for these variants, they do not seem to instigate a heightened inflammatory response in these cell types.

The evaluation of cytopathogenicity furnished an intriguing revelation. The B.1 variant displayed a greater capacity to induce cell death in mononuclear cells and neutrophils compared to the P.1 variant. This characteristic might hint at the B.1 variant’s potential to inflict greater tissue damage, although this must be contextualized within the broader physiological scenario. Despite this, absolute cell counts post-infection were comparable for both variants, suggesting that while the B.1 variant may inflict greater initial cell damage, it does not necessarily lead to a higher cell death rate over time.

In terms of viral replication within macrophage cells, our findings align with prior research which posits an absence of productive replication within mononuclear cells [54,55,56]. Neither the P.1 nor B.1 variants appeared to have acquired an enhanced ability to replicate within these cells. However, these cells were obtained from patients with severe COVID-19, and perhaps, unlike newly recruited mononuclear phagocytes or neutrophils, they may already be overactivated or even unresponsive in senescence. Some in vivo and in vitro studies show that these cells, when in contact with the virus, acquire an abnormal, senescent and even immunosuppressive phenotype [61,64,65,66].

Similar studies corroborate and complement our analyses. A study using a hamster intranasal infection model showed that the replication, infectivity and pathogenicity of the P.1 variant is similar to the two original strains of SARS-CoV-2 [55]. However, this work showed that the P.1 variant can infect the upper respiratory tract cells of mice, which the original strain cannot. As a result, damage to the lung tissue in these mice was more severe [55]. The evidence also shows an epidemiological outcome distinct from the P.1 variant. Through mathematical models, Naveca FG et al. suggest that p.1 is known for its high virulence. The recent European Center for Diseases and Prevention (ECDC) study confirms its increased risk of hospitalization (2.6 times higher) and ICU admission (2.2 times higher) [47]. Official data relates 559,000 deaths due to the P.1 variant, but this number could be even higher [67].

The in-depth study of the spike protein has revealed subtle differences that might have functional implications. This protein, central to the virus’s entry into human cells, is paramount for devising treatment and vaccination strategies [10,11,22,23,35,36,37]. Alterations in this protein can affect how well the virus binds to cells, influencing its infectiousness [22,23]. Our meticulous analysis of several spike protein versions hints at potential differences in their binding abilities, but confirming these suspicions would require functional tests.

Swapping an amino acid within a protein’s structure can recalibrate its properties, determining its interactions with other entities, such as the ACE-2 receptor or antibodies. We have observed that certain mutations in the spike protein can recalibrate its bond with the ACE-2 receptor, tweaking the strength of the bond, as seen with the N501Y mutation. Similarly, mutations such as E484K can change how the protein interacts with antibodies, possibly allowing the virus to dodge the body’s defenses more effectively [18,19].

The existence cycle of SARS-CoV-2 hinges on its spike protein’s bond with the human ACE-2 receptor, a relationship corroborated by past research. This bond’s fundamental role indicates that any spike protein alterations, especially within the RBD, can significantly modulate the virus’s infectious properties. Turoňová and colleagues have illustrated the spike protein’s inherent flexibility, its protective glycans and dynamic behaviors, offering foundational knowledge for our investigations.

Figure 5 in our report provides a deep dive into the structural differences across the spike protein forms. By observing it from various angles, we gain a richer understanding of where mutations occur and their possible implications on the efficiency of ACE-2 binding. Combining these visuals with an investigation into the protein’s electric and hydrophobic traits equips us with a powerful toolkit for analysis.

A standout observation was the SARS-CoV-2 spike protein’s remarkable adaptability. Merging the insights from Turoňová’s team about RBD alignments with our own, it is apparent that certain protein structures favor enhanced cell interactions. This insight paints a clearer picture of the virus’s adaptability and evolution. A focal point of our exploration, represented in Figure 6, is the categorization based on the spike proteins’ inherent traits. Predecessor versions, such as WT, B.1.1.28 and P.1, showcased overlapping features hinting at a mutual ancestry. This method of classification highlights the contrasts between the foundational and more recent strains, aiding our grasp of viral evolution and possible hurdles in treatment. Highlighting the spike proteins based on their electric properties, especially noting the similarity between P.1.7 and P.1.8, offers a roadmap of the virus’s evolution. Keeping tabs on these variants is crucial, especially to identify any increased infectiousness or heightened resistance to the body’s defenses.

## 5. Limitations

Our research focuses on the B.1 and P.1 variants of SARS-CoV-2, but there are several other emerging variants that also require investigation due to their potential unique interactions and effects. A significant limitation is the lack of healthy donors to serve as controls and the absence of an answer to the question of how the new mutations affect the pathogenesis of these variants.

## 6. Conclusions

In conclusion, the impact of the SARS-CoV-2 variants (B.1 and P.1) on cytokine profiles and cell damage does not significantly intensify the cytokine storm associated with severe disease, and the B.1 variant exhibits heightened initial cell damage. Furthermore, in-depth analysis of the spike protein emphasized its pivotal role in viral infectiousness and adaptability, with certain mutations potentially enhancing the virus’s interaction with human cells or evading immunity. This research underscores the importance of ongoing surveillance of these variants and continuous examination of the spike protein’s modifications for creating informed therapeutic and vaccine strategies.

## Data Availability

The data are contained within this article.

## Supplementary Materials

The following supporting information can be downloaded at: https://www.mdpi.com/article/10.3390/microorganisms12040720/s1, Supplementary Figure S1: Epogram result of webPIPSA analysis. Result of electrostatic potential analysis of the surfaces of the analyzed models and distances between the models evidenced in the matrix. The S glycoproteins of the Wt-Wuhan and B.1.1.28 variants are the ones with the greatest distances in relation to the others. P1 S protein models. and P.1.3 and P.1.8 and P1.4 have smaller distances between them. The P.1.7 S protein has a smaller distance in relation to the S protein of the Gamma variant than in relation to the ones from the ancestral lineage B.1.1.28 and WT-Wuhan (Wild-type).
