# Supplementary material for: A Comparative Analysis of Innate Immune Responses and the Structural Characterization of Spike from SARS-CoV-2 Gamma Variants and Subvariants
Source: Microorganisms. 2024 Apr 2;12(4):720. doi: 10.3390/microorganisms12040720 (PMC11052025; doi:10.3390/microorganisms12040720)
Supplement: Supplementary file 1 [file microorganisms-12-00720-s001.zip › microorganisms-2622876-supplementary.pdf]

Supplementary Materials

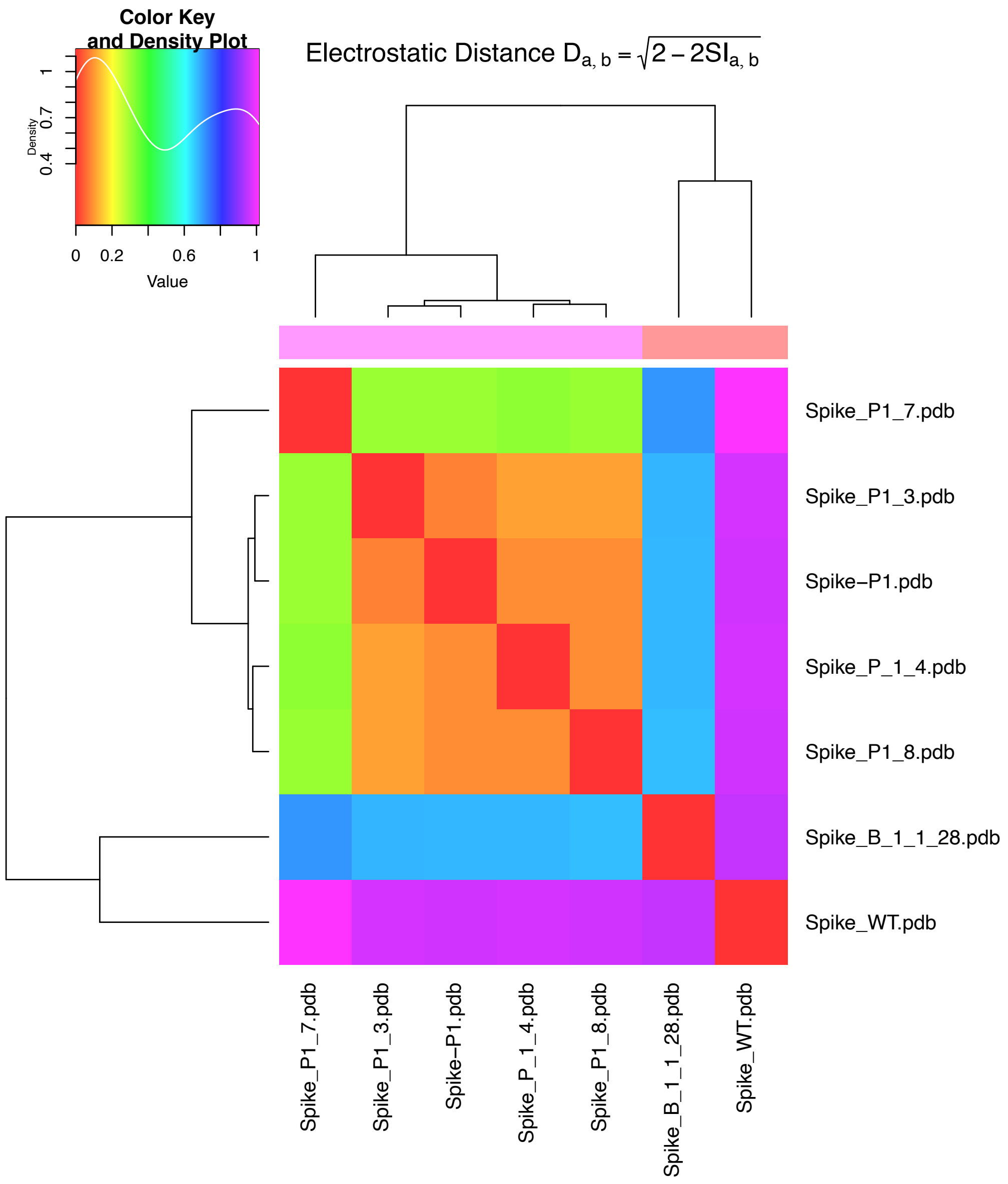

Figure S1. Epogram result of webPIPSA analysis. Result of electrostatic potential analysis of the surfaces of the analyzed models and distances between the models evidenced in the matrix. The S glycoproteins of the Wt-Wuhan and B.1.1.28 variants are the ones with the greatest distances in relation to the others. P1 S protein models. and P.1.3 and P.1.8 and P1.4 have smaller distances between them. The P.1.7 S protein has a smaller distance in relation to the S protein of the Gamma variant than in relation to the ones from the ancestral lineage B.1.1.28 and WT-Wuhan (Wild-type).
